# *N*-Benzyl-2-chloro­quinazolin-4-amine

**DOI:** 10.1107/S1600536814006746

**Published:** 2014-04-16

**Authors:** Tarek Mohamed, Abdeljalil Assoud, Praveen P. N. Rao

**Affiliations:** a Department of Chemistry, University of Waterloo, 200 University Ave. W, Waterloo, Ontario N2L 3G1, Canada; b School of Pharmacy, Health Sciences Campus, University of Waterloo, 200 University Ave. W, Waterloo, Ontario N2L 3G1, Canada

## Abstract

The asymmetric unit of the title compound, C_15_H_12_ClN_3_, contains two independent mol­ecules. The quinazoline ring system in each is essentially planar, with maximum deviations of 0.025 (16) and 0.0171 (16) Å. The dihedral angles between quinazoline ring systems and the phenyl rings are 88.25 (8) and 85.28 (16)° in the two independent mol­ecules. In the crystal, alternating independent mol­ecules are linked by N—H⋯N hydrogen bonds, forming chains along [001].

## Related literature   

For the biological activity of some quinazoline and related derivatives, see: Deng & Mani (2006 ▶); Lee *et al.* (1995 ▶); Lopez *et al.* (2011 ▶); Mohamed *et al.* (2011 ▶); Wynne *et al.* (2009 ▶); Yoshida & Taguchi (1992 ▶); Zhang *et al.* (2009 ▶); Zhou *et al.* (2011 ▶).
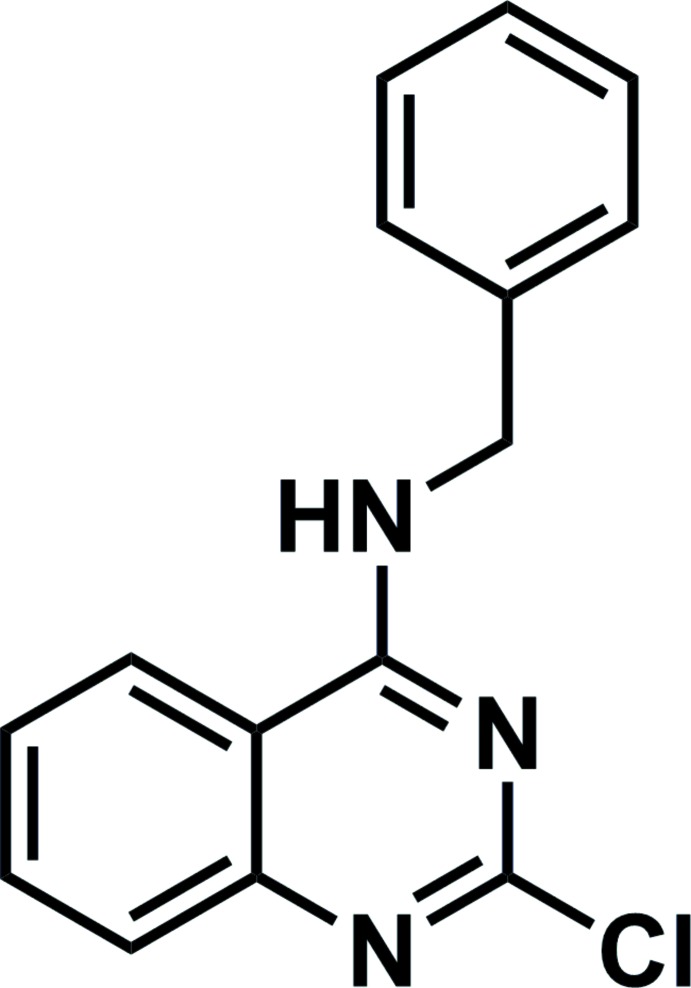


## Experimental   

### 

#### Crystal data  

C_15_H_12_ClN_3_
*M* *_r_* = 269.73
Triclinic, 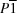

*a* = 9.4018 (1) Å
*b* = 13.0108 (1) Å
*c* = 13.3035 (1) Å
α = 113.968 (1)°
β = 105.377 (1)°
γ = 100.213 (1)°
*V* = 1356.69 (2) Å^3^
*Z* = 4
Mo *K*α radiation
μ = 0.27 mm^−1^
*T* = 296 K
0.35 × 0.26 × 0.10 mm

#### Data collection  

Bruker Kappa APEXII diffractometer
Absorption correction: multi-scan (*SADABS*; Bruker, 2008 ▶) *T*_min_ = 0.911, *T*_max_ = 0.974
22349 measured reflections
6531 independent reflections
5274 reflections with *I* > 2σ(*I*)
*R*_int_ = 0.024
3 standard reflections every 15 min intensity decay: none

#### Refinement  

*R*[*F* ^2^ > 2σ(*F* ^2^)] = 0.039
*wR*(*F* ^2^) = 0.086
*S* = 1.13
6531 reflections
344 parameters
H-atom parameters constrained
Δρ_max_ = 0.33 e Å^−3^
Δρ_min_ = −0.49 e Å^−3^

### 

Data collection: *APEX2* (Bruker, 2008 ▶); cell refinement: *SAINT* (Bruker, 2008 ▶); data reduction: *SAINT*; program(s) used to solve structure: *SHELXS97* (Sheldrick, 2008 ▶); program(s) used to refine structure: *SHELXL2013* (Sheldrick, 2008 ▶); molecular graphics: *DIAMOND* (Brandenburg, 1999 ▶); software used to prepare material for publication: *publCIF* (Westrip, 2010 ▶).

## Supplementary Material

Crystal structure: contains datablock(s) I. DOI: 10.1107/S1600536814006746/lh5690sup1.cif

Structure factors: contains datablock(s) I. DOI: 10.1107/S1600536814006746/lh5690Isup2.hkl

Supporting information file. DOI: 10.1107/S1600536814006746/lh5690Isup3.cml

CCDC reference: 993912

Additional supporting information:  crystallographic information; 3D view; checkCIF report

## Figures and Tables

**Table 1:** Hydrogen-bond geometry (Å, °)

*D*—H⋯*A*	*D*—H	H⋯*A*	*D*⋯*A*	*D*—H⋯*A*
N3*A*—H3*AA*⋯N1*B* ^i^	0.86	2.21	2.9954 (17)	152
N3*B*—H3*BA*⋯N1*A*	0.86	2.18	2.9482 (16)	149

Symmetry code: (i) 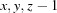.

**Fig. 1.**
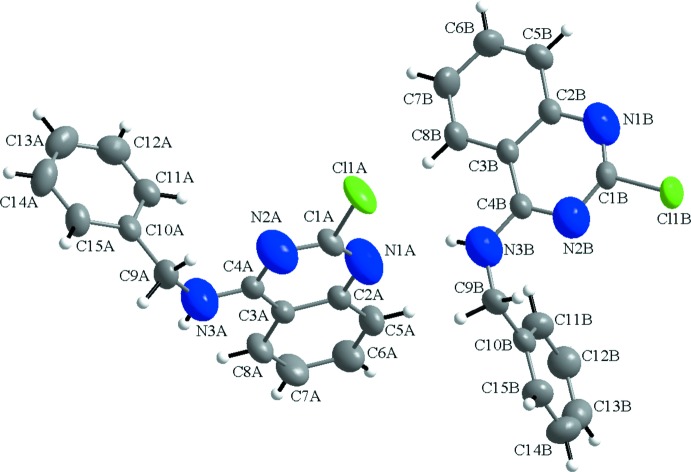
The asymmetric unit of (I) with displacement ellipsoids drawn at the 50% probability level.

**Fig. 2.**
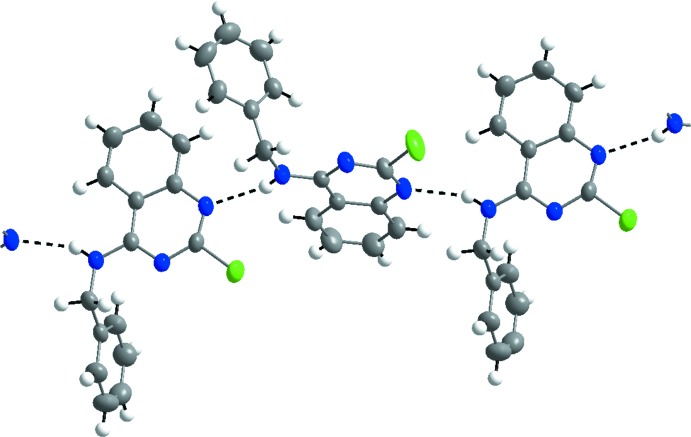
Part of the crystal structure showing hydrogen bonds (dashed lines) which connect molecules along [001].

## supplementary crystallographic information

### 1. Introduction

Quinazolines represent an important class of nitro­gen containing heterocycles that are present in a number of therapeutically useful small molecules such as tyrosine kinase inhibitors (Zhang *et al.*, 2009; Lopez *et al.*, 2011) and adrenergic antagonists (Zhou *et al.*, 2011). In our study, we have used 2,4-di­chloro­quinazolines to explore the selectivity of nucleophilic displacement of halogens (Mohamed *et al.*, 2011). It is known that the monocyclic compound 2,4-di­chloro­pyrimidine, generally favours C-4 substitution selectively or as the major regioisomer during simple S_N_Ar reactions with amine nucleophiles (Deng *et al.*, 2006). A similar effect is seen with the bicyclic 2,4-di­chloro­quinazoline (Yoshida *et al.*, 1992). Reaction of 2,4-di­chloro­quinazoline with benzyl­amine provided selective substitution at C-4 position. The chemical structure was confirmed by determining the crystal structure of *N*-benzyl-2-chloro­quinazoline-4-amine (I).

### 2. Experimental

The title compound was prepared by slowly adding 0.75 mL of benzyl­amine (6.53mmol) to a mixture of 2,4-di­chloro­quinazoline (1g, 5.02 mmol) in 20 mL of methanol on ice-bath while stirring. The solution was allowed to stir on ice for 5 minutes before drop wise addition of DIPEA (1.75 mL, 10.04 mmol). The reaction was allowed to stir on the ice-bath for an additional 5 minutes and then refluxed at 348–353K for 3 hours. After cooling to 298 K, the solvent was evaporated in vacuo and the residue was re-dissolved in EtOAc, washed with saturated NaHCO_3_ and NaCl solution (2 x 15 mL) respectively. Aqueous layer (pH 7.5-8.0) was washed with EtOAc (1 x 15 mL) and the combined organic layer was dried over anhydrous MgSO_4_, then filtered. The organic layer was evaporated in vacuo and the resulting yellowish solid was further purified by silica gel column chromatography using EtOAc:MeOH (5:1) as eluent to afford the desired product as a white solid (1.02 g, 75% yield). Mp: 442–444K. ^1^H-NMR (300MHz, DMSO-d_6_) d 9.24 (t, 6.0Hz 1H), d 8.27 (d, *J* = 6.0, 1H), d 7.75 (t, *J* = 9.0Hz, 1H), d 7.59 (d, *J* = 6.0, 1H), d 7.49 (t, *J* = 9.0Hz, 1H), d 7.20-7.34 (m, 5H), d 4.72 (d, *J* = 6.0, 2H). ESI-MS *m/z*: 270 [M+1]^+^ (Lee *et al.*, 1995; Wynne *et al.*, 2009). Crystal growth was carried out by dissolving 5mg of the product in 20mL of ethanol at room temperature and heating the solution to 353K to form a concentrated solution with a reduced volume of 10mL. The hot solution was transferred to a scintillation vial, capped and stored at 275-279K undisturbed for four days at which needle-like crystals were obtained which were suitable for X-ray diffraction.

### 2.1. Refinement

All H-atoms were positioned in geometrically idealized positions and refined using a riding model with C—H = 0.93–1.00 Å and N—H = 0.86 Å and isotropic displacement parameters of U_iso_(H) = 1.2U_eq_(C).

### 3. Results and discussion

The asymmetric unit of (I) is shown in Fig. 1. The quinazoline ring system [N1/N2/C1–8] in each independent molecule is essentially planar with maximum deviations of 0.025 (16) and 0.0171 (16) Å for C4A and C4B, repectively. The dihedral angles between quinazoline ring systems and the phenyl rings [C10–C15 ] are 88.25 (8)° and 85.28 (16) ° for molecules A and B, respectively. In the crystal, alternating independent molecules are linked by N—H···N hydrogen bonds forming chains along [001] (Fig .2).

### Crystal data

**Table:**

C_15_H_12_ClN_3_	*Z* = 4
*M**_r_* = 269.73	*F*(000) = 560
Triclinic, *P*1	*D*_x_ = 1.321 Mg m^−^^3^
*a* = 9.4018 (1) Å	Mo *K*α radiation, λ = 0.71073 Å
*b* = 13.0108 (1) Å	Cell parameters from 407 reflections
*c* = 13.3035 (1) Å	θ = 1.5–30°
α = 113.968 (1)°	µ = 0.27 mm^−^^1^
β = 105.377 (1)°	*T* = 296 K
γ = 100.213 (1)°	Block, colourless
*V* = 1356.69 (2) Å^3^	0.35 × 0.26 × 0.10 mm

### Data collection

**Table:**

Bruker Kappa APEXII diffractometer	5274 reflections with *I* > 2σ(*I*)
Radiation source: fine-focus sealed tube	*R*_int_ = 0.024
Graphite monochromator	θ_max_ = 28.0°, θ_min_ = 2.4°
ω and φ scans	*h* = −12→12
Absorption correction: multi-scan (*SADABS*; Bruker, 2008)	*k* = −17→17
*T*_min_ = 0.911, *T*_max_ = 0.974	*l* = −17→17
22349 measured reflections	3 standard reflections every 15 min
6531 independent reflections	intensity decay: none

### Refinement

**Table:**

Refinement on *F*^2^	Hydrogen site location: inferred from neighbouring sites
Least-squares matrix: full	H-atom parameters constrained
*R*[*F*^2^ > 2σ(*F*^2^)] = 0.039	*w* = 1/[σ^2^(*F*_o_^2^) + (0.0141*P*)^2^ + 0.4615*P*] where *P* = (*F*_o_^2^ + 2*F*_c_^2^)/3
*wR*(*F*^2^) = 0.086	(Δ/σ)_max_ < 0.001
*S* = 1.13	Δρ_max_ = 0.33 e Å^−^^3^
6531 reflections	Δρ_min_ = −0.49 e Å^−^^3^
344 parameters	Extinction correction: *SHELXL2013* (Sheldrick, 2008), Fc^*^=kFc[1+0.001xFc^2^λ^3^/sin(2θ)]^-1/4^
0 restraints	Extinction coefficient: 0.0041 (6)

### Special details

**Table:**

Geometry. All e.s.d.'s (except the e.s.d. in the dihedral angle between two l.s. planes) are estimated using the full covariance matrix. The cell e.s.d.'s are taken into account individually in the estimation of e.s.d.'s in distances, angles and torsion angles; correlations between e.s.d.'s in cell parameters are only used when they are defined by crystal symmetry. An approximate (isotropic) treatment of cell e.s.d.'s is used for estimating e.s.d.'s involving l.s. planes.

### Fractional atomic coordinates and isotropic or equivalent isotropic displacement parameters (Å^2^)

**Table:**

	*x*	*y*	*z*	*U*_iso_*/*U*_eq_	
Cl1A	0.80906 (7)	0.05252 (4)	0.09218 (4)	0.07177 (16)	
N1A	0.75236 (15)	0.23972 (11)	0.09405 (10)	0.0431 (3)	
N2A	0.81249 (15)	0.11531 (11)	−0.06785 (10)	0.0437 (3)	
N3A	0.81842 (15)	0.15522 (12)	−0.21892 (11)	0.0461 (3)	
H3AA	0.8049	0.1991	−0.2522	0.055*	
C1A	0.78763 (18)	0.14847 (13)	0.03282 (13)	0.0432 (3)	
C2A	0.74116 (16)	0.31670 (12)	0.04724 (12)	0.0381 (3)	
C3A	0.76545 (16)	0.29524 (13)	−0.05817 (12)	0.0373 (3)	
C4A	0.79930 (16)	0.18703 (13)	−0.11613 (12)	0.0375 (3)	
C5A	0.7048 (2)	0.41884 (15)	0.10729 (14)	0.0515 (4)	
H5AA	0.6880	0.4334	0.1769	0.062*	
C6A	0.6941 (2)	0.49701 (16)	0.06391 (16)	0.0617 (5)	
H6AA	0.6697	0.5645	0.1042	0.074*	
C7A	0.7193 (2)	0.47677 (16)	−0.04001 (17)	0.0648 (5)	
H7AA	0.7121	0.5309	−0.0685	0.078*	
C8A	0.7548 (2)	0.37788 (15)	−0.10035 (15)	0.0532 (4)	
H8AA	0.7718	0.3651	−0.1696	0.064*	
C9A	0.86081 (18)	0.05090 (14)	−0.27836 (13)	0.0485 (4)	
H9AA	0.8007	0.0127	−0.3629	0.058*	
H9AB	0.8337	−0.0050	−0.2502	0.058*	
C10A	1.03177 (17)	0.07992 (12)	−0.25780 (12)	0.0387 (3)	
C11A	1.14458 (19)	0.16829 (14)	−0.15033 (14)	0.0472 (4)	
H11A	1.1148	0.2127	−0.0895	0.057*	
C12A	1.3005 (2)	0.19105 (16)	−0.13271 (17)	0.0594 (4)	
H12A	1.3753	0.2502	−0.0600	0.071*	
C13A	1.3458 (2)	0.12649 (18)	−0.2225 (2)	0.0670 (5)	
H13A	1.4510	0.1419	−0.2106	0.080*	
C14A	1.2353 (2)	0.03958 (16)	−0.32935 (19)	0.0638 (5)	
H14A	1.2656	−0.0037	−0.3903	0.077*	
C15A	1.0797 (2)	0.01593 (14)	−0.34694 (15)	0.0499 (4)	
H15A	1.0057	−0.0438	−0.4197	0.060*	
Cl1B	0.49469 (5)	0.17163 (4)	0.57865 (4)	0.06102 (13)	
N1B	0.78003 (14)	0.23433 (11)	0.59600 (10)	0.0413 (3)	
N2B	0.58069 (14)	0.20758 (11)	0.42357 (10)	0.0416 (3)	
N3B	0.63353 (14)	0.23214 (12)	0.27581 (10)	0.0442 (3)	
H3BA	0.7001	0.2522	0.2480	0.053*	
C1B	0.63823 (17)	0.20955 (13)	0.52713 (13)	0.0402 (3)	
C2B	0.89177 (16)	0.26517 (12)	0.55393 (12)	0.0378 (3)	
C3B	0.85120 (16)	0.26871 (12)	0.44613 (12)	0.0369 (3)	
C4B	0.68648 (16)	0.23599 (12)	0.38078 (12)	0.0375 (3)	
C5B	1.04922 (18)	0.29186 (14)	0.62032 (14)	0.0487 (4)	
H5BA	1.0771	0.2897	0.6918	0.058*	
C6B	1.16159 (19)	0.32102 (16)	0.58031 (15)	0.0569 (4)	
H6BA	1.2657	0.3381	0.6246	0.068*	
C7B	1.12207 (19)	0.32550 (17)	0.47386 (16)	0.0593 (4)	
H7BA	1.1997	0.3457	0.4477	0.071*	
C8B	0.96948 (18)	0.30032 (15)	0.40786 (14)	0.0497 (4)	
H8BA	0.9438	0.3041	0.3372	0.060*	
C9B	0.46910 (17)	0.19581 (14)	0.20594 (13)	0.0460 (3)	
H9BA	0.4136	0.1313	0.2143	0.055*	
H9BB	0.4553	0.1652	0.1230	0.055*	
C10B	0.39638 (16)	0.29301 (13)	0.23906 (12)	0.0404 (3)	
C11B	0.48004 (19)	0.40835 (15)	0.32662 (15)	0.0537 (4)	
H11B	0.5860	0.4282	0.3680	0.064*	
C12B	0.4087 (2)	0.49486 (16)	0.35374 (17)	0.0625 (5)	
H12B	0.4667	0.5721	0.4134	0.075*	
C13B	0.2534 (2)	0.46736 (18)	0.29325 (18)	0.0626 (5)	
H13B	0.2054	0.5255	0.3112	0.075*	
C14B	0.1692 (2)	0.35318 (19)	0.20577 (19)	0.0679 (5)	
H14B	0.0635	0.3341	0.1641	0.081*	
C15B	0.23929 (19)	0.26642 (16)	0.17885 (15)	0.0558 (4)	
H15B	0.1803	0.1892	0.1196	0.067*	

### Atomic displacement parameters (Å^2^)

**Table:**

	*U*^11^	*U*^22^	*U*^33^	*U*^12^	*U*^13^	*U*^23^
Cl1A	0.1269 (4)	0.0514 (3)	0.0519 (3)	0.0322 (3)	0.0343 (3)	0.0364 (2)
N1A	0.0559 (7)	0.0427 (7)	0.0340 (6)	0.0137 (6)	0.0193 (6)	0.0209 (5)
N2A	0.0582 (8)	0.0414 (7)	0.0353 (6)	0.0188 (6)	0.0178 (6)	0.0205 (5)
N3A	0.0578 (8)	0.0574 (8)	0.0391 (7)	0.0293 (6)	0.0251 (6)	0.0288 (6)
C1A	0.0559 (9)	0.0386 (8)	0.0358 (7)	0.0107 (7)	0.0141 (7)	0.0222 (6)
C2A	0.0399 (7)	0.0396 (7)	0.0334 (7)	0.0112 (6)	0.0126 (6)	0.0177 (6)
C3A	0.0387 (7)	0.0416 (8)	0.0347 (7)	0.0133 (6)	0.0131 (6)	0.0214 (6)
C4A	0.0373 (7)	0.0440 (8)	0.0330 (7)	0.0132 (6)	0.0127 (6)	0.0202 (6)
C5A	0.0650 (10)	0.0511 (9)	0.0413 (8)	0.0237 (8)	0.0250 (8)	0.0198 (7)
C6A	0.0832 (13)	0.0488 (10)	0.0561 (10)	0.0336 (9)	0.0268 (9)	0.0224 (8)
C7A	0.0954 (14)	0.0545 (10)	0.0622 (11)	0.0365 (10)	0.0306 (10)	0.0382 (9)
C8A	0.0746 (11)	0.0542 (10)	0.0464 (9)	0.0274 (9)	0.0275 (8)	0.0325 (8)
C9A	0.0537 (9)	0.0500 (9)	0.0367 (8)	0.0168 (7)	0.0194 (7)	0.0144 (7)
C10A	0.0517 (8)	0.0360 (7)	0.0358 (7)	0.0169 (6)	0.0196 (6)	0.0207 (6)
C11A	0.0579 (9)	0.0452 (9)	0.0392 (8)	0.0178 (7)	0.0177 (7)	0.0205 (7)
C12A	0.0550 (10)	0.0531 (10)	0.0601 (11)	0.0078 (8)	0.0105 (8)	0.0289 (9)
C13A	0.0535 (10)	0.0669 (12)	0.0961 (15)	0.0193 (9)	0.0366 (11)	0.0475 (12)
C14A	0.0730 (12)	0.0543 (10)	0.0812 (13)	0.0246 (9)	0.0521 (11)	0.0317 (10)
C15A	0.0645 (10)	0.0394 (8)	0.0473 (9)	0.0147 (7)	0.0300 (8)	0.0172 (7)
Cl1B	0.0539 (2)	0.0861 (3)	0.0579 (3)	0.0173 (2)	0.0323 (2)	0.0430 (2)
N1B	0.0468 (7)	0.0482 (7)	0.0335 (6)	0.0145 (6)	0.0176 (5)	0.0224 (6)
N2B	0.0424 (6)	0.0507 (7)	0.0378 (6)	0.0165 (6)	0.0186 (5)	0.0238 (6)
N3B	0.0442 (7)	0.0593 (8)	0.0366 (6)	0.0178 (6)	0.0175 (5)	0.0279 (6)
C1B	0.0465 (8)	0.0433 (8)	0.0391 (7)	0.0146 (6)	0.0240 (6)	0.0222 (6)
C2B	0.0445 (8)	0.0363 (7)	0.0333 (7)	0.0129 (6)	0.0161 (6)	0.0164 (6)
C3B	0.0410 (7)	0.0376 (7)	0.0328 (7)	0.0117 (6)	0.0158 (6)	0.0167 (6)
C4B	0.0455 (8)	0.0381 (7)	0.0334 (7)	0.0160 (6)	0.0176 (6)	0.0183 (6)
C5B	0.0473 (8)	0.0550 (9)	0.0383 (8)	0.0116 (7)	0.0094 (7)	0.0235 (7)
C6B	0.0391 (8)	0.0670 (11)	0.0533 (10)	0.0079 (8)	0.0096 (7)	0.0271 (9)
C7B	0.0439 (9)	0.0745 (12)	0.0571 (10)	0.0067 (8)	0.0222 (8)	0.0324 (9)
C8B	0.0483 (9)	0.0615 (10)	0.0413 (8)	0.0104 (7)	0.0189 (7)	0.0280 (8)
C9B	0.0469 (8)	0.0510 (9)	0.0336 (7)	0.0127 (7)	0.0099 (6)	0.0188 (7)
C10B	0.0418 (7)	0.0489 (8)	0.0320 (7)	0.0108 (6)	0.0131 (6)	0.0228 (6)
C11B	0.0460 (9)	0.0503 (9)	0.0505 (9)	0.0099 (7)	0.0080 (7)	0.0198 (8)
C12B	0.0681 (12)	0.0483 (10)	0.0621 (11)	0.0160 (9)	0.0205 (9)	0.0219 (9)
C13B	0.0703 (12)	0.0638 (12)	0.0747 (12)	0.0340 (10)	0.0371 (10)	0.0409 (10)
C14B	0.0452 (9)	0.0793 (14)	0.0774 (13)	0.0228 (9)	0.0166 (9)	0.0387 (11)
C15B	0.0447 (9)	0.0570 (10)	0.0501 (9)	0.0092 (7)	0.0088 (7)	0.0199 (8)

### Geometric parameters (Å, º)

**Table:**

Cl1A—C1A	1.7403 (14)	Cl1B—C1B	1.7503 (14)
N1A—C1A	1.2962 (19)	N1B—C1B	1.2964 (19)
N1A—C2A	1.3826 (18)	N1B—C2B	1.3866 (17)
N2A—C1A	1.3291 (18)	N2B—C1B	1.3275 (18)
N2A—C4A	1.3359 (18)	N2B—C4B	1.3383 (17)
N3A—C4A	1.3305 (17)	N3B—C4B	1.3289 (17)
N3A—C9A	1.4537 (19)	N3B—C9B	1.4489 (18)
N3A—H3AA	0.8600	N3B—H3BA	0.8600
C2A—C5A	1.401 (2)	C2B—C5B	1.403 (2)
C2A—C3A	1.4040 (18)	C2B—C3B	1.4051 (19)
C3A—C8A	1.407 (2)	C3B—C8B	1.4047 (19)
C3A—C4A	1.444 (2)	C3B—C4B	1.4446 (19)
C5A—C6A	1.364 (2)	C5B—C6B	1.365 (2)
C5A—H5AA	0.9300	C5B—H5BA	0.9300
C6A—C7A	1.392 (3)	C6B—C7B	1.394 (2)
C6A—H6AA	0.9300	C6B—H6BA	0.9300
C7A—C8A	1.366 (2)	C7B—C8B	1.367 (2)
C7A—H7AA	0.9300	C7B—H7BA	0.9300
C8A—H8AA	0.9300	C8B—H8BA	0.9300
C9A—C10A	1.506 (2)	C9B—C10B	1.508 (2)
C9A—H9AA	0.9700	C9B—H9BA	0.9700
C9A—H9AB	0.9700	C9B—H9BB	0.9700
C10A—C15A	1.384 (2)	C10B—C11B	1.379 (2)
C10A—C11A	1.385 (2)	C10B—C15B	1.382 (2)
C11A—C12A	1.378 (2)	C11B—C12B	1.383 (2)
C11A—H11A	0.9300	C11B—H11B	0.9300
C12A—C13A	1.377 (3)	C12B—C13B	1.365 (3)
C12A—H12A	0.9300	C12B—H12B	0.9300
C13A—C14A	1.368 (3)	C13B—C14B	1.370 (3)
C13A—H13A	0.9300	C13B—H13B	0.9300
C14A—C15A	1.375 (2)	C14B—C15B	1.378 (3)
C14A—H14A	0.9300	C14B—H14B	0.9300
C15A—H15A	0.9300	C15B—H15B	0.9300
			
C1A—N1A—C2A	114.20 (12)	C1B—N1B—C2B	113.92 (12)
C1A—N2A—C4A	115.75 (12)	C1B—N2B—C4B	115.30 (12)
C4A—N3A—C9A	124.03 (13)	C4B—N3B—C9B	123.18 (12)
C4A—N3A—H3AA	118.0	C4B—N3B—H3BA	118.4
C9A—N3A—H3AA	118.0	C9B—N3B—H3BA	118.4
N1A—C1A—N2A	131.20 (13)	N1B—C1B—N2B	131.77 (13)
N1A—C1A—Cl1A	115.28 (11)	N1B—C1B—Cl1B	114.95 (10)
N2A—C1A—Cl1A	113.52 (11)	N2B—C1B—Cl1B	113.29 (11)
N1A—C2A—C5A	118.76 (13)	N1B—C2B—C5B	118.87 (13)
N1A—C2A—C3A	121.78 (13)	N1B—C2B—C3B	121.78 (13)
C5A—C2A—C3A	119.46 (13)	C5B—C2B—C3B	119.35 (13)
C2A—C3A—C8A	119.10 (13)	C8B—C3B—C2B	119.24 (13)
C2A—C3A—C4A	116.21 (12)	C8B—C3B—C4B	124.64 (13)
C8A—C3A—C4A	124.69 (13)	C2B—C3B—C4B	116.11 (12)
N3A—C4A—N2A	117.73 (13)	N3B—C4B—N2B	117.24 (13)
N3A—C4A—C3A	121.47 (13)	N3B—C4B—C3B	121.66 (12)
N2A—C4A—C3A	120.80 (12)	N2B—C4B—C3B	121.10 (12)
C6A—C5A—C2A	120.19 (15)	C6B—C5B—C2B	120.14 (14)
C6A—C5A—H5AA	119.9	C6B—C5B—H5BA	119.9
C2A—C5A—H5AA	119.9	C2B—C5B—H5BA	119.9
C5A—C6A—C7A	120.63 (16)	C5B—C6B—C7B	120.74 (15)
C5A—C6A—H6AA	119.7	C5B—C6B—H6BA	119.6
C7A—C6A—H6AA	119.7	C7B—C6B—H6BA	119.6
C8A—C7A—C6A	120.38 (16)	C8B—C7B—C6B	120.19 (15)
C8A—C7A—H7AA	119.8	C8B—C7B—H7BA	119.9
C6A—C7A—H7AA	119.8	C6B—C7B—H7BA	119.9
C7A—C8A—C3A	120.23 (15)	C7B—C8B—C3B	120.34 (14)
C7A—C8A—H8AA	119.9	C7B—C8B—H8BA	119.8
C3A—C8A—H8AA	119.9	C3B—C8B—H8BA	119.8
N3A—C9A—C10A	112.90 (13)	N3B—C9B—C10B	114.71 (12)
N3A—C9A—H9AA	109.0	N3B—C9B—H9BA	108.6
C10A—C9A—H9AA	109.0	C10B—C9B—H9BA	108.6
N3A—C9A—H9AB	109.0	N3B—C9B—H9BB	108.6
C10A—C9A—H9AB	109.0	C10B—C9B—H9BB	108.6
H9AA—C9A—H9AB	107.8	H9BA—C9B—H9BB	107.6
C15A—C10A—C11A	118.33 (14)	C11B—C10B—C15B	118.09 (15)
C15A—C10A—C9A	119.91 (14)	C11B—C10B—C9B	122.94 (14)
C11A—C10A—C9A	121.75 (13)	C15B—C10B—C9B	118.97 (14)
C12A—C11A—C10A	120.64 (15)	C10B—C11B—C12B	120.99 (16)
C12A—C11A—H11A	119.7	C10B—C11B—H11B	119.5
C10A—C11A—H11A	119.7	C12B—C11B—H11B	119.5
C13A—C12A—C11A	120.16 (17)	C13B—C12B—C11B	120.26 (17)
C13A—C12A—H12A	119.9	C13B—C12B—H12B	119.9
C11A—C12A—H12A	119.9	C11B—C12B—H12B	119.9
C14A—C13A—C12A	119.70 (17)	C12B—C13B—C14B	119.29 (17)
C14A—C13A—H13A	120.1	C12B—C13B—H13B	120.4
C12A—C13A—H13A	120.1	C14B—C13B—H13B	120.4
C13A—C14A—C15A	120.30 (17)	C13B—C14B—C15B	120.74 (17)
C13A—C14A—H14A	119.9	C13B—C14B—H14B	119.6
C15A—C14A—H14A	119.8	C15B—C14B—H14B	119.6
C14A—C15A—C10A	120.86 (16)	C14B—C15B—C10B	120.61 (17)
C14A—C15A—H15A	119.6	C14B—C15B—H15B	119.7
C10A—C15A—H15A	119.6	C10B—C15B—H15B	119.7
			
C2A—N1A—C1A—N2A	−1.2 (2)	C2B—N1B—C1B—N2B	0.5 (2)
C2A—N1A—C1A—Cl1A	177.92 (10)	C2B—N1B—C1B—Cl1B	−179.43 (10)
C4A—N2A—C1A—N1A	−0.1 (3)	C4B—N2B—C1B—N1B	0.1 (2)
C4A—N2A—C1A—Cl1A	−179.20 (11)	C4B—N2B—C1B—Cl1B	−179.97 (10)
C1A—N1A—C2A—C5A	−179.46 (14)	C1B—N1B—C2B—C5B	−178.94 (14)
C1A—N1A—C2A—C3A	0.4 (2)	C1B—N1B—C2B—C3B	0.2 (2)
N1A—C2A—C3A—C8A	−179.03 (14)	N1B—C2B—C3B—C8B	−179.85 (14)
C5A—C2A—C3A—C8A	0.8 (2)	C5B—C2B—C3B—C8B	−0.8 (2)
N1A—C2A—C3A—C4A	1.3 (2)	N1B—C2B—C3B—C4B	−1.2 (2)
C5A—C2A—C3A—C4A	−178.80 (13)	C5B—C2B—C3B—C4B	177.84 (13)
C9A—N3A—C4A—N2A	−3.4 (2)	C9B—N3B—C4B—N2B	−1.8 (2)
C9A—N3A—C4A—C3A	176.58 (13)	C9B—N3B—C4B—C3B	177.81 (13)
C1A—N2A—C4A—N3A	−177.93 (13)	C1B—N2B—C4B—N3B	178.31 (13)
C1A—N2A—C4A—C3A	2.1 (2)	C1B—N2B—C4B—C3B	−1.3 (2)
C2A—C3A—C4A—N3A	177.36 (13)	C8B—C3B—C4B—N3B	0.8 (2)
C8A—C3A—C4A—N3A	−2.3 (2)	C2B—C3B—C4B—N3B	−177.75 (13)
C2A—C3A—C4A—N2A	−2.6 (2)	C8B—C3B—C4B—N2B	−179.62 (14)
C8A—C3A—C4A—N2A	177.74 (15)	C2B—C3B—C4B—N2B	1.9 (2)
N1A—C2A—C5A—C6A	179.50 (15)	N1B—C2B—C5B—C6B	179.16 (15)
C3A—C2A—C5A—C6A	−0.4 (2)	C3B—C2B—C5B—C6B	0.0 (2)
C2A—C5A—C6A—C7A	−0.2 (3)	C2B—C5B—C6B—C7B	0.4 (3)
C5A—C6A—C7A—C8A	0.2 (3)	C5B—C6B—C7B—C8B	−0.2 (3)
C6A—C7A—C8A—C3A	0.2 (3)	C6B—C7B—C8B—C3B	−0.6 (3)
C2A—C3A—C8A—C7A	−0.8 (2)	C2B—C3B—C8B—C7B	1.0 (2)
C4A—C3A—C8A—C7A	178.83 (16)	C4B—C3B—C8B—C7B	−177.45 (16)
C4A—N3A—C9A—C10A	−99.54 (17)	C4B—N3B—C9B—C10B	83.98 (18)
N3A—C9A—C10A—C15A	−145.39 (14)	N3B—C9B—C10B—C11B	1.7 (2)
N3A—C9A—C10A—C11A	35.9 (2)	N3B—C9B—C10B—C15B	−178.82 (14)
C15A—C10A—C11A—C12A	−0.5 (2)	C15B—C10B—C11B—C12B	0.2 (2)
C9A—C10A—C11A—C12A	178.29 (15)	C9B—C10B—C11B—C12B	179.71 (15)
C10A—C11A—C12A—C13A	0.5 (3)	C10B—C11B—C12B—C13B	−0.4 (3)
C11A—C12A—C13A—C14A	0.0 (3)	C11B—C12B—C13B—C14B	0.2 (3)
C12A—C13A—C14A—C15A	−0.5 (3)	C12B—C13B—C14B—C15B	0.2 (3)
C13A—C14A—C15A—C10A	0.6 (3)	C13B—C14B—C15B—C10B	−0.4 (3)
C11A—C10A—C15A—C14A	−0.1 (2)	C11B—C10B—C15B—C14B	0.2 (2)
C9A—C10A—C15A—C14A	−178.87 (15)	C9B—C10B—C15B—C14B	−179.30 (16)

### Hydrogen-bond geometry (Å, º)

**Table:**

*D*—H···*A*	*D*—H	H···*A*	*D*···*A*	*D*—H···*A*
N3*A*—H3*AA*···N1*B*^i^	0.86	2.21	2.9954 (17)	152
N3*B*—H3*BA*···N1*A*	0.86	2.18	2.9482 (16)	149

Symmetry code: (i) *x*, *y*, *z*−1.

## References

[bb1] Brandenburg, K. (1999). *DIAMOND* Crystal Impact GbR, Bonn, Germany.

[bb2] Bruker (2008). *APEX2* and *SAINT* Bruker AXS Inc., Madison, Wisconsin, USA.

[bb3] Deng, X. & Mani, N. S. (2006). *Org. Lett.* **8**, 269–272. 10.1021/ol052663x 16408892

[bb4] Lee, S. J., Konishi, Y., Yu, D. T., Miskowski, T. A., Riviello, C. M., Macina, O. T., Frierson, M. R., Kondo, K., Sugitani, M., Sircar, J. C. & Balzejewski, K. M. (1995). *J. Med. Chem.* **38**, 3547–3557. 10.1021/jm00018a014 7658441

[bb5] Lopez, C. O., Garcia, C. A., Nunez, M. C., Kimatrai, M., Rubino, G. M. E., Morales, F., Perez, G. V. & Campos, J. M. (2011). *Curr. Med. Chem.* **18**, 943–963. 10.2174/092986711794940824 21254978

[bb6] Mohamed, T., Zhao, X., Habib, L. K., Yang, J. & Rao, P. P. N. (2011). *Bioorg. Med. Chem.* **19**, 2269–2281. 10.1016/j.bmc.2011.02.030 PMC3066269 21429752

[bb7] Sheldrick, G. M. (2008). *Acta Cryst.* A**64**, 112–122. 10.1107/S0108767307043930 18156677

[bb8] Westrip, S. P. (2010). *J. Appl. Cryst.* **43**, 920–925.

[bb9] Wynne, G. M., De Moor, O., Johnson, P. D. & Vickers, R. (2009). World Patent WO 2009/001060 A2.

[bb10] Yoshida, K. & Taguchi, M. (1992). *J. Chem. Soc. Perkin Trans. 1*, pp. 919–922.

[bb11] Zhang, J., Yang, P. L. & Gray, N. S. (2009). *Nat. Rev. Cancer*, **9**, 28–39. 10.1038/nrc2559 PMC12406740 19104514

[bb12] Zhou, G., Wang, L., Ma, Y., Wang, L., Zhang, Y. & Jiang, W. (2011). *Bioorg. Med. Chem. Lett.* **21**, 5905–5909. 10.1016/j.bmcl.2011.07.122 21875797

